# Do cholesterol levels and continuity of statin use affect colorectal cancer incidence in older adults under 75 years of age?

**DOI:** 10.1371/journal.pone.0250716

**Published:** 2021-04-23

**Authors:** Kyu-Tae Han, Seungju Kim

**Affiliations:** 1 Division of Cancer Control and Policy, National Cancer Control Institute, National Cancer Center, Goyang, Republic of Korea; 2 Department of Nursing, College of Nursing, The Catholic University of Korea, Seoul, Republic of Korea; Universidad Miguel Hernandez de Elche, SPAIN

## Abstract

**Introduction:**

Colorectal cancer(CRC) is 3rd most common cancer and has a relatively high mortality rate. Currently, the relationships between CRC and serum cholesterol or statin treatment, especially in older adults under 75 years of age, remain questionable due to a lack of data. The present study evaluated the association between serum cholesterol levels and statin treatment continuity and CRC risk in older adults under 75 years of age.

**Methods:**

This study used senior cohort data obtained from the National Health Insurance Service of South Korea. The selected cohort contains 131,266 participants who were enrolled from 2009 to 2011 and followed for up to 5 years. Serum cholesterol levels were classified as categorical variables, and continuity of statin treatment was evaluated based on dyslipidemia diagnosis and average medication possession ratio. We used Cox regression analysis to evaluate the associations between CRC risk and serum cholesterol level or statin use.

**Results:**

A low level of high-density lipoprotein cholesterol(HDL-C) was significantly associated with high CRC risk compared to an HDL-C level in the normal range(hazard ratio [HR]: 1.197, 95% CI: 1.040–1.377). A high level of low-density lipoprotein cholesterol(LDL-C) was associated with increased CRC risk compared to a normal LDL-C level, but not statistically significant. Statin use was associated with decreased CRC risk, and high medication compliance was inversely associated with CRC risk in patients with and without dyslipidemia.

**Conclusions:**

Statin use was associated with decreased CRC risk, and high medication compliance was inversely associated with CRC risk in patients with and without dyslipidemia compared to non-use of medication. Regular health examinations can help identify individuals who are vulnerable to CRC, and continued statin use may be associated with a reduced risk of CRC. This is particularly important in patients with diabetes and dyslipidemia.

## Introduction

Colorectal cancer (CRC) is the third most common cancer and ranks as the second most common cause of cancer-related death worldwide [1]. Physical activity, diet, individual behavioral factors, and obesity are associated with CRC risk [2, 3], and dyslipidemia and abnormal glucose metabolism are positively associated with CRC risk [4, 5]. However, the involvement of individual serum cholesterol levels and continuity of statin therapy in CRC risk remain unknown [6, 7].

In 2017, the incidence of CRC in Korea was 29.7 per 100,000 individuals, making it the third most common cancer in the country after thyroid and stomach cancer [8]. CRC incidence rates vary according to age and sex, and CRC is the most common cancer in females and fourth most common cancer in males aged 65 years and older. Early detection and removal of cancer is important to improving patient care outcomes, and in the United States, regular CRC screenings are recommended for vulnerable populations and individuals over the age of 50 up to 75 years of age [9]. In Korea, the National Cancer Screening Program recommends CRC screening through fecal occult blood tests for male and female aged 50 years or older [10]. However, the CRC screening rate in Korea was 25% in 2012, the lowest among the five major cancers, and remained below 40% in 2015 [10, 11]. Thus, other approaches to CRC detection or prevention based on an understanding of the disease beyond CRC screening may be needed.

Serum cholesterol levels are hypothesized to be markers of tumor progression [12], but the relationship between serum cholesterol levels and CRC is unclear. Studies have shown that low-density lipoprotein cholesterol (LDL-C) is associated with CRC progression [13], high-density lipoprotein cholesterol (HDL-C) is inversely related to CRC risk [14], and total cholesterol (TC) and triglyceride (TG) levels are positively associated with increased CRC risk [5]. In the United States, a reduction in serum TC prior to cancer diagnosis was associated with an increased risk of CRC diagnosis, and this risk was higher in non-statin users [7]. Similarly, meta-analyses have shown that statin use decreases CRC risk [15] and that pre-diagnostic statin use is weakly associated with decreased CRC risk, although direct evidence for these effects, particularly after cancer diagnosis, is lacking [16]. A study conducted in individuals between 18 and 90 years of age, statin use did not affect CRC risk [17]. A case-control study did not found that the use of statin was associated with a reduced risk of CRC [18]. The effect of statin use on CRC risk remains controversial, and more evidence is needed to understand the relationship between continued use of statins and CRC. Notably, older adults have a higher incidence of CRC than the general population. Because serum cholesterol levels and statin use in older adults may also differ from the general population, further study of the relationship between CRC and these factors is critical to understanding age-related differences in CRC incidence.

In this study, we evaluated how serum cholesterol levels and continuity of statin use affect CRC risk. We classified serum cholesterol levels based on the diagnosis criteria for dyslipidemia. We also assessed patient diagnosis with dyslipidemia, which is closely related to statin use, and categorized participants with dyslipidemia into three groups based on 5-year average statin adherence. Finally, we conducted a subgroup analysis of the effects of serum cholesterol levels and statin use on CRC risk in patients with and without diabetes.

## Methods and materials

### Database and data collection

This study used senior cohort data from the National Health Insurance Service of South Korea. This cohort included a baseline population of 558,147 participants aged 60 years and older in 2002 who were followed through 2015 [19]. These data include personal demographic information, medical treatment data, health examination records, and hospital characteristics. National health examinations occurred biennially and included self-reported questionnaires (e.g., lifestyle, past medical history, and family medical history) and biometric information (e.g., serum cholesterol level and blood pressure). Medical data for all participants were included in insurance claim data, including diagnoses, visit dates, and costs.

For our analysis, we considered data collected from 232,968 participants enrolled in the cohort from 2009 to 2011. For each patient, we considered data obtained during a 5-year observation period that began on the date of their first health examination between 2009 and 2011. We excluded any participants who had been diagnosed with any type of cancer from 2002 to 2008. Additionally, patients lacking records of health examination items (e.g., serum cholesterol levels and body mass index [BMI]) were excluded. Next, we excluded patients who were diagnosed with CRC (International Classification of Diseases [ICD]-10 code: C18-C20) prior to the date of their first health examination from 2009 to 2011. Participants over 75 years of age are excluded as of the baseline year of participation from 2009 to 2011. Finally, patients with metabolic disease prior to 2009 or who were prescribed medication of Fibrates and Ezetimibe other than statins that affect plasma cholesterol levels were excluded. Participants were observed until 2015, taking into account the follow-up period of each patient. Ultimately, 131,266 participants were included in the study and followed up during the study period (S1 Appendix).

### Variables

The outcome variable was the onset of CRC, which we identified by ICD-10 code (C18-C20). The start date was the first date of health examination during the study period, and the end date was the date of CRC diagnosis, date of death, or the end date of the study period. First, we set each participant’s observation period to 5 years based on the start date. For example, the observation period of a participant who underwent a health examination on February 1, 2009, ended on January 31, 2014, and the observation period of a participant who received a health examination on December 1, 2011, ended on December 31, 2015 (the end date of the study period). Second, we evaluated the onset of CRC in each participant during the observation period and defined the first diagnosis date as the end date. Since we defined CRC based on the ICD-10 codes, there may be diagnostic errors in the course of outpatient treatment. To reduce potential diagnostic errors in claim data, we evaluated whether the patient was hospitalized in the same or the next year with the same major diagnosis based on the first diagnosis date of CRC. Finally, we defined a “CRC case” as a patient who was diagnosed with CRC and was hospitalized with the same major diagnosis.

The variables of interest in this study were serum cholesterol levels and continuity of statin use. Serum cholesterol levels were categorized according to the 2018 Korean Dyslipidemia Management guidelines [20] as follows: LDL-C (normal, <130 mg/dL; moderate, 130–159 mg/dL; high, ≥160 mg/dL), HDL-C (low, <40 mg/dL; normal, 40–59 mg/dL; high, ≥60 mg/dL), TC (normal, <200 mg/dL; high, ≥200 mg/dL), and TG (normal, <150 mg/dL; high, ≥150 mg/dL). Statin use was based on a prescription with a medication code and was measured as the sum of the total number of days of prescription for each patient. We calculated medication possession ratio (MPR) for each patient per year by dividing the number of days the patient was supplied with statin (≤365) by 365 days [21]. Next, we calculated the average MPR during the 5-year observation period and categorized participants into three groups based on medication compliance, including none (0%), poor (<50%), and high compliance (≥50%). Dyslipidemia is defined as a patient diagnosed with dyslipidemia based on the ICD-10 code (E78). Finally, participants were classified into six categories, taking into account whether they were diagnosed with dyslipidemia and using statins.

Covariates included the following demographic and individual behavioral factors: sex (male or female); age; family history of cancer (yes, unknown, or no); BMI; smoker status (current, ex-smoker, or non-smoker); drinking days per week; insurance (Medicaid, self-employed, or employee); residential area (capital area, metropolitan, or other); and diabetes (yes or no, according to ICD-10 codes E10-E14).

### Ethical considerations

The data we use is secondary data and all of the patient’s personal data is encrypted and difficult to identify. This study was approved for waiver from the Institutional Review Board, Eulji University (IRB number: EUIRB2020-025).

### Statistical analysis

The distribution of each categorical variable was examined by an analysis of frequencies and percentages, and χ^2^ tests were performed. For continuous variables, t-tests were performed to compare mean and standard deviation values. All variables were entered simultaneously into the fully adjusted model. Cox-proportional hazard ratio (HR) was used to identify serum cholesterol levels and continuity of statin use associated with CRC risk while controlling for potential confounding variables. We conducted subgroup analyses to evaluate associations between serum cholesterol level and continuity of statin use and CRC relative to diabetes diagnosis. All statistical analyses were performed using the SAS statistical software version 9.4 (SAS Institute, Cary, NC, USA). A p-value of <0.05 was considered statistically significant.

## Results

The baseline characteristics of study participants are shown in Table 1. The study included 131,266 participants enrolled from 2009 to 2011, of whom 1,552 (1.2%) were diagnosed with CRC. The incidence of CRC was higher among participants with low HDL-C levels (<40 mg/dL; 1.5% incidence) than among participants with normal (130–159 mg/dL; 1.2%) or high (≥160 mg/dL; 1.1%) HDL-C levels (p = 0.0005). We found no significant associations between serum cholesterol levels and CRC incidence for the other serum cholesterol levels evaluated (LDL-C, TC, and TG). We observed a statistically significant difference in CRC incidence based on statin use, with the lowest CRC incidence being observed in patients with dyslipidemia and high medication compliance (Dyslipidemia Yes, MPR ≥50%; 0.8%, p<0.0001). Of the demographic characteristics evaluated, CRC incidence was higher in males than in females, and BMI, smoking, drinking, and residual area were related to CRC incidence.

**Table 1:** Study participants grouped by baseline characteristics.

Characteristic	Colorectal Cancer Cases^a^			Total Participants^b^
	Yes	No	p-value	
**HDL-C**				
Low (<40 mg/dL)	272 (1.5)	18,353 (98.5)	0.0005	18,625 (14.2)
Normal (40–59 mg/dL)	881 (1.2)	75,123 (98.8)		76,004 (57.9)
High (≥60 mg/dL)	399 (1.1)	36,238 (98.9)		36,637 (27.9)
**LDL-C**				
Low (<130 mg/dL)	1,047 (1.2)	85,472 (98.8)	0.2990	86,519 (65.9)
Moderate (130–159 mg/dL)	329 (1.1)	29,640 (98.9)		29,969 (22.8)
High (≥160 mg/dL)	176 (1.2)	14,602 (98.8)		14,778 (11.3)
**TC**				
Low (<200 mg/dL)	891 (1.2)	71,488 (98.8)	0.0745	72,379 (55.1)
High (≥200 mg/dL)	661 (1.1)	58,226 (98.9)		58,887 (44.9)
**TG**				
Low (<150 mg/dL)	1,025 (1.2)	87,750 (98.9)	0.1881	88,775 (67.6)
High (≥150 mg/dL)	527 (1.2)	41,964 (98.8)		42,491 (32.4)
**Dyslipidemia diagnosis (MPR)**				
Yes (0%)	110 (1.0)	10,404 (99.0)	<.0001	10,514 (8.0)
Yes (<50%)	106 (1.0)	10,833 (99.0)		10,939 (8.3)
Yes (≥50%)	117 (0.8)	13,921 (99.2)		14,038 (10.7)
No (0%)	939 (1.3)	70,191 (98.7)		71,130 (54.2)
No (<50%)	124 (1.3)	9,608 (98.7)		9,732 (7.4)
No (≥50%)	156 (1.0)	14,757 (99.9)		14,913 (11.4)
**Sex**				
Male	926 (1.6)	58,771 (98.4)	<.0001	59,697 (45.5)
Female	626 (0.9)	70,943 (99.1)		71,569 (54.5)
**Age (years)**	70.75 ± 2.26	70.57 ± 2.29	0.0023	70.57 ± 2.29
**Family history of cancer**				
Yes	134 (1.3)	9,931 (98.7)	0.3527	10,065 (7.7)
Unknown	641 (1.2)	53,967 (98.8)		54,608 (41.6)
No	777 (1.2)	65,816 (98.8)		66,593 (50.7)
**Diabetes**				
Yes	537 (1.2)	46,088 (98.8)	0.4628	46,625 (35.5)
No	1,015 (1.2)	83,626 (98.8)		84,641 (64.5)
**Insurance**				
Medicaid	6 (1.1)	548 (98.9)	0.4004	554 (0.4)
Self-Employed	496 (1.2)	39,408 (98.8)		39,904 (30.4)
Employees	1,050 (1.2)	89,758 (98.8)		90,808 (69.2)
**Residential area**				
Capital area	627 (1.3)	48,651 (98.7)	0.0417	49,278 (37.5)
Metropolitan	355 (1.2)	29,869 (98.8)		30,224 (23.0)
Other	570 (1.1)	51,194 (98.9)		51,764 (39.4)
**BMI**	24.10 ± 2.99	23.92 ± 3.11	0.0195	23.92 ± 3.11
**Smoke**				
Current	236 (1.4)	16,817 (98.6)	<.0001	17,053 (13.0)
Ex-smoker	304 (1.6)	18,291 (98.4)		18,595 (14.2)
Non-smoker	1,012 (1.1)	94,606 (98.9)		95,618 (72.8)
**Drinking frequency (days per week)**	2.17 ± 2.06	1.77 ± 1.71	<.0001	1.78 ± 1.71
**Total**	1,552 (1.2)	129,714 (98.8)		131,266 (100.0)

HDL-C, high-density lipoprotein cholesterol; LDL-C, low-density lipoprotein cholesterol; TC, total cholesterol; TG, triglyceride; MPR, medication possession ratio; BMI, body mass index.

^a^Unit: participants per outcome (% of group) or average value ± SD.

^b^Unit: participants per group (% of all participants).

Table 2 shows the results of the Cox models of the associations between serum cholesterol levels and statin use and the CRC risk. All variables were entered simultaneously into the fully adjusted model. Low HDL-C was significantly associated with high CRC risk compared to HDL-C values in the normal range (HR: 1.197, 95% CI: 1.057–1.374), and high HDL-C was associated with low CRC risk, although this association was not statistically significant. High LDL-C was associated with increased CRC risk (HR: 1.219, 95% CI: 0.993–1.496) compared to LDL-C values in the normal range, but not statistically significant. No significant associations were found between TC or TG and CRC risk. Statin use was significantly associated with CRC risk. High medication compliance was inversely associated with CRC risk in patients without dyslipidemia compared to non-medication users, although this association was only statistically significant for individuals with an MPR ≥50% (HR: 0.799, 95% CI: 0.672–0.950). Older adults diagnosed with dyslipidemia had a lower CRC risk than non-dyslipidemia patients without statins, but the magnitude of this effect differed based on MPR (MPR 0%, HR: 0.786, p = 0.0177; MPR <50%, HR: 0.769, p = 0.0132; MPR ≥50%: HR: 0.671, p<0.0001).

**Table 2:** The association between serum cholesterol levels and colorectal cancer.

	HR	95% CI		p-value
**HDL-C**				
Low (<40 mg/dL)	1.197	1.040	1.377	0.0122
Normal (40–59 mg/dL)	1.000	-	-	
High (≥60 mg/dL)	0.968	0.856	1.096	0.6108
**LDL-C**				
Normal (<130 mg/dL)	1.000	-	-	
Moderate (130–159 mg/dL)	1.004	0.849	1.187	0.9638
High (≥160 mg/dL)	1.219	0.993	1.496	0.0589
**TC**				
Normal (<200 mg/dL)	1.000	-	-	
High (≥200 mg/dL)	0.993	0.851	1.159	0.9293
**TG**				
Normal (<150 mg/dL)	1.000	-	-	
High (≥150 mg/dL)	1.058	0.944	1.186	0.3335
**Dyslipidemia diagnosis (MPR)**				
Yes (0%)	0.786	0.644	0.959	0.0177
Yes (<50%)	0.769	0.625	0.947	0.0132
Yes (≥50%)	0.671	0.551	0.818	<.0001
No (0%)	1.000	-	-	
No (<50%)	0.976	0.807	1.180	0.8015
No (≥50%)	0.799	0.672	0.950	0.0109
**Sex**				
Male	1.635	1.436	1.862	<.0001
Female	1.000	-	-	
**Age**	1.042	1.020	1.065	0.0002
**Family history of cancer**				
Yes	1.195	0.994	1.436	0.0574
Unknown	1.000	0.901	1.111	0.9950
No	1.000	-	-	
**Diabetes**				
Yes	0.971	0.872	1.081	0.5932
No	1.000	-	-	
**BMI**	1.035	1.018	1.053	<.0001
**Smoke**				
Current	0.902	0.769	1.058	0.2060
Ex-smoker	1.035	0.893	1.200	0.6443
Non-smoker	1.000	-	-	
**Drinking days per week**	1.067	1.039	1.096	<.0001
**Insurance**				
Medicaid	1.016	0.455	2.267	0.9690
Self-Employed	1.100	0.988	1.225	0.0821
Employees	1.000	-	-	
**Residential area**				
Capital area	1.173	1.045	1.316	0.0066
Metropolitan	1.083	0.948	1.237	0.2429
Other	1.000	-	-	

HR, hazard ratio; 95% CI, 95% confidence interval; HDL-C, high-density lipoprotein cholesterol; LDL-C, low-density lipoprotein cholesterol; TC, total cholesterol; TG, triglyceride; MPR, medication possession ratio. All variables were entered simultaneously.

**Fig 1:**
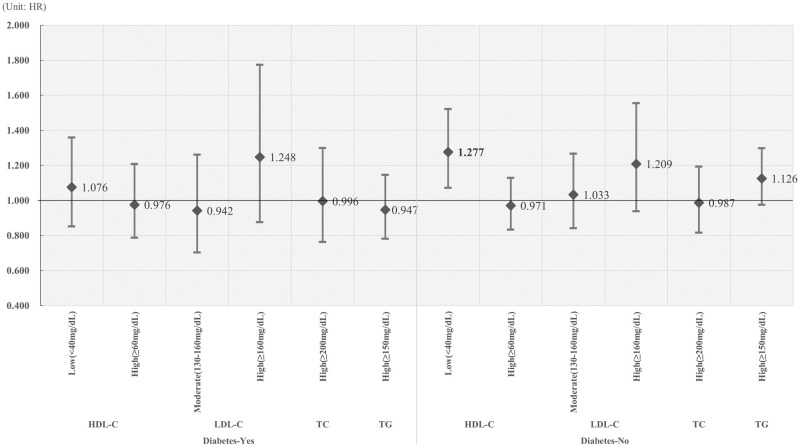
The association between serum cholesterol levels and CRC risk in patients with or without diabetes. Bold is statistically significant.

**Fig 2:**
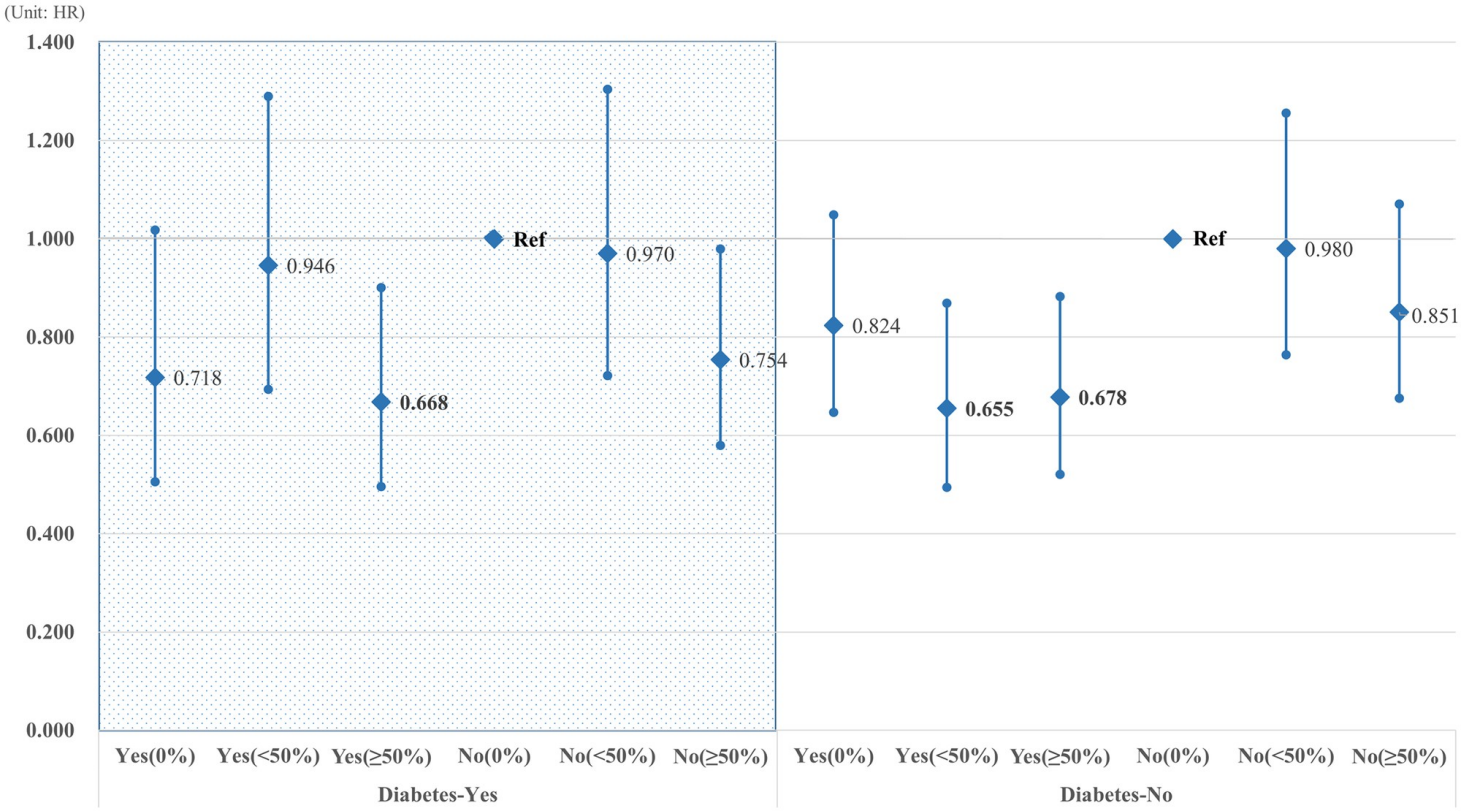
The association between statin use and CRC risk in patients with or without diabetes. Bold is statistically significant.

Fig 1 shows the association between serum cholesterol level and CRC in patients with or without diabetes. In patients without diabetes, low HDL-C was significantly associated with an increased risk of CRC (HR: 1.277, 95% CI: 1.072–1.522). Fig 2 shows the association between statin use and CRC risk in patients with or without diabetes. In participants with diabetes, dyslipidemia patients with high medication compliance had a significantly lower risk of developing CRC than those with non-dyslipidemia who did not take statins (HR: 0.668, 95% CI: 0.496–0.901). In participants without diabetes, we observed results similar to the main findings of this study and low CRC risk in patients with dyslipidemia and high medication compliance (MPR <50%, HR: 0.655, p = 0.0034; MPR ≥50%, HR: 0.678, p = 0.0039).

## Discussion

Serum cholesterol is important for cell membrane formations, and changes in cholesterol levels can affect cancer development [12]. Although various studies have been conducted on the involvement of cholesterol in cancer, it remains unknown whether cholesterol levels and the use of statins affect an individual’s risk of developing cancer. This study evaluated the associations between CRC risk and serum cholesterol levels and continuity of statin use. In this study, we found that low HDL-C levels were associated with an increased risk of developing CRC. Statin use was associated with a decreased incidence of CRC, especially in patients with dyslipidemia, and the size of this apparent protective effect was largest in patients with high medication compliance.

Our results are similar to the results of a previous study that reported that serum HDL-C concentration is inversely associated with the risk of colon cancer [5, 14]. However, unlike previous studies that showed that high TG and TC levels are associated with increased CRC risk [5], we did not find a significant association between TG or TC and CRC risk in the present study. In our present study, high TC was inversely associated with the risk of CRC; although, the difference was not statistically significant. Similar results were found in a Korean study and in a Finnish study, which found a high TC (≥240mg/dL) was associated with a decreased risk of colon cancer and rectal cancer, respectively [22, 23]. These results suggest that TC levels may affect cancer sub-sites, such as colon and rectal, differently. An Italian study found that high TC and LDL-C levels are associated with increased CRC risk in adults, especially in males [24]. Another study found that metabolic syndrome is associated with CRC risk, especially in adults, and that low HDL-C levels are associated with high CRC risk [25]. Although one meta-analysis showed that statin use moderately reduces CRC risk [15], another meta-analysis found significant results only in cohort and case-control studies [26].

Although many studies have investigated the relationship between serum cholesterol levels and CRC, the mechanism underlying the effect of cholesterol on CRC risk remains unclear. One proposal, which is related to cholesterol metabolism, suggests that high serum cholesterol levels may promote CRC by increasing the production of cholesterol-based bile acids [27, 28]. Statins inhibit 3-hydroxy-3 methylglutaryl-coenzyme A reductase (HMG-CoA reductase) and affect cholesterol biosynthesis. In addition, genetic errors in cells can affect regulation of apoptosis, which can lead to CRC [6]. The use of statins can induce cell apoptosis, resulting in cell death, which can affect the onset of CRC. Therefore, regulation of serum cholesterol by statin therapy may have a protective effect against the development of CRC. In particular, this study found that continuity of statin use had a greater protective effect on CRC in both patients with dyslipidemia and in older adults without dyslipidemia. Interestingly, in our study, patients with dyslipidemia who were not on statin therapy exhibited a reduced risk of CRC. According to the guidelines for dyslipidemia treatment, patients diagnosed with dyslipidemia begin treatment based on their LDL-C level, and statins are recommended for patients with high LDL-C [20]. Therefore, a patient diagnosed with dyslipidemia, without high LDL-C levels, is likely to be well-managed cholesterol levels, which may reduce their CRC risk. Taken together, these results suggest that control of LDL-C is important in older adults under 75 years of age, and statin therapy in particular is effective in patients with high drug compliance.

In our subgroup analysis of diabetes patients, we found that greater continuity of statin use was associated with a decreased incidence of CRC in both diabetic and non-diabetic patients and that this effect was statistically significant in patients with dyslipidemia. Further, the magnitude of this effect was greatest in patients diagnosed with diabetes and dyslipidemia who received statin therapy and exhibited high medication compliance. This result suggests that continuous statin use may be an effective method of preventing CRC, especially in older adults diagnosed with both diabetes and dyslipidemia. In addition, in older adults without diabetes, low HDL-C was significantly associated with increased CRC risk, which suggests that HDL-C levels should be regularly evaluated in this patient population.

Although CRC incidence in Korea is high, the CRC screening rate is low, which may be related to limited access to screening. Whereas fecal-based screening is not highly accessible to the general population, a CRC screening test based on measuring serum cholesterol levels could improve access to screening and increase early detection of vulnerable patients. In addition, statin therapy may be effective in preventing CRC, and our results suggest that continued statin use in older adults under 75 years of age may be an important strategy to reduce their risk of CRC. Thus, healthcare providers must educate their patients and recognize that continued statin use is associated with a reduced CRC risk, especially for patients with both diabetes and dyslipidemia. Finally, the relationship between statin use and serum cholesterol levels and CRC is still controversial, and further studies of the mechanisms involved are needed.

This study has several limitations. First, CRC is associated with several risk factors, and individual behavioral factors that were not measured in the present study may influence the CRC risk. To reduce such errors, we considered individual factors that may influence CRC and included family cancer history in our analysis. Further, changes in serum cholesterol during the study period may affect the onset of CRC; therefore, further analysis will be needed to consider time-dependent covariates. Second, because we used claim data to calculate MPR based on medications prescribed, the actual patient medication intake may differ from our calculated MPR, which could affect our results. However, because we calculated the average MPR over the entire 5-year follow-up period, the effects of these differences will be not large. Third, because our study excluded patients who did have results from the health examination, which may affect our study results. Finally, because this study did not classify cancer subtypes, such as colon or rectal cancer, further research is needed for specific cancer types. Despite these limitations, this study provides evidence that continuity of statin use and regular measurement of serum cholesterol levels, including LDL-C and HDL-C, are important for the health of older adults under the age of 75. The data presented in this study will help identify patients who are vulnerable to CRC among older adults.

Cancer requires active treatment, and various approaches should be considered for early detection of cancer. This study provides evidence that high LDL-C and low HDL-C levels are associated with increased CRC risk and that statin treatment decreases CRC risk in older adults under 75 years of age. In patients with dyslipidemia and diabetes in particular, continuity of statin use can contribute significantly to lowering CRC risk. Therefore, in vulnerable and high-risk populations, regular health examinations, including evaluation of serum cholesterol levels, will be important to prevent the development of CRC.

## Supporting information

S1 Appendix Flow diagram of study population. (DOCX)

## Abbreviations

CRC: colorectal cancer
HDL-C: high-density lipoprotein cholesterol
HR: hazard ratio
ICD: International Classification of Diseases
LDL-C: low-density lipoprotein cholesterol
MPR: medication possession ratio
TC: total cholesterol
TG: triglyceride
