# Supplementary material for: Do cholesterol levels and continuity of statin use affect colorectal cancer incidence in older adults under 75 years of age?
Source: PLoS One. 2021 Apr 23;16(4):e0250716. doi: 10.1371/journal.pone.0250716 (PMC8064530; doi:10.1371/journal.pone.0250716)
Supplement: S1 Appendix — (DOCX) [file pone.0250716.s001.docx]

**S1 Appendix**


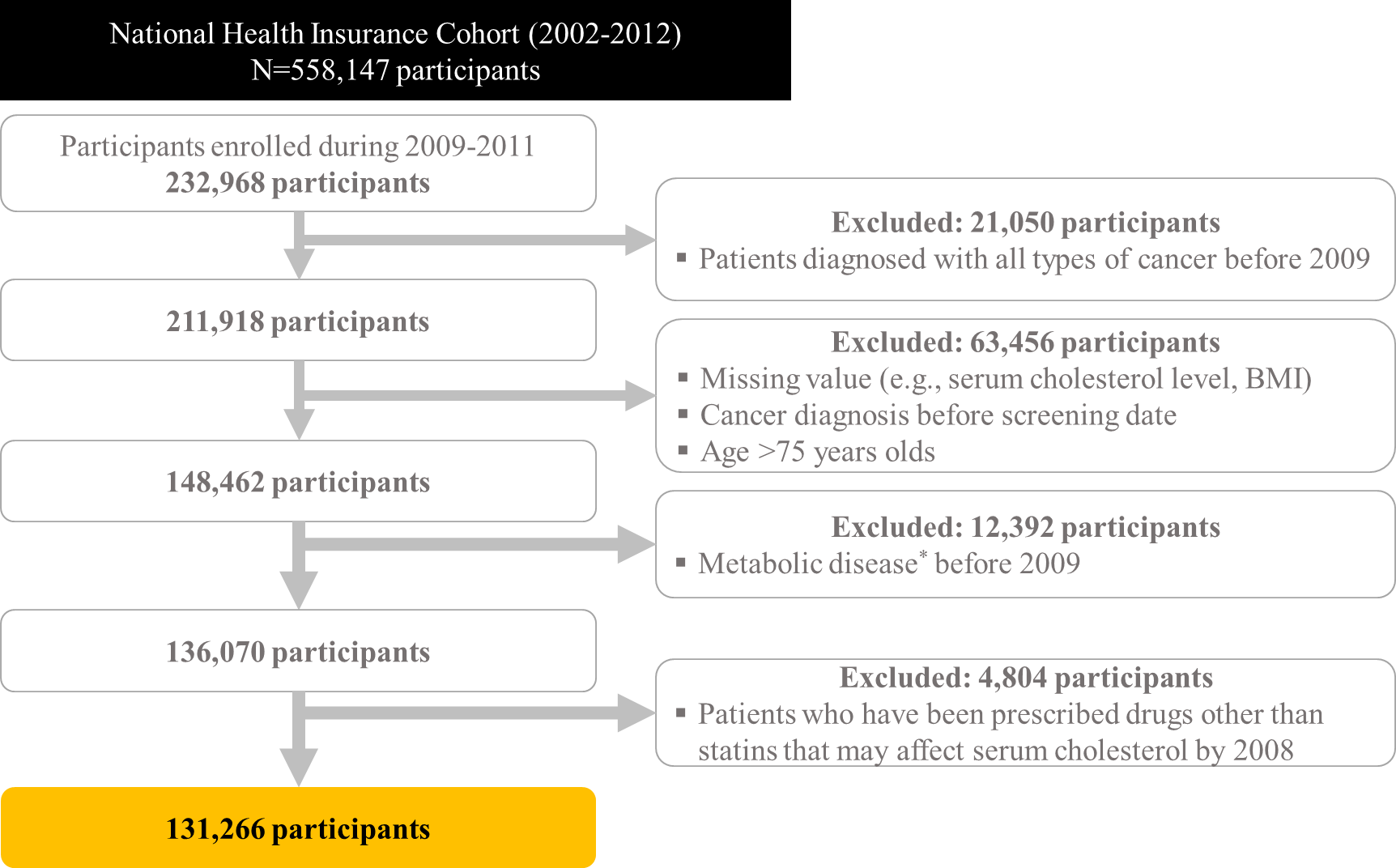


**S1 Appendix. Flow diagram of study population**

*Metabolic disease was defined as the presence of three or more of the following criteria: Abdominal obesity(Male≥ 90cm/ Female≥ 85cm), High blood pressure(Systolic≥ 130mmHg or diastolic≥ 85mmHg or diagnosis of hypertension), High fasting blood glucose concentration(fasting glucose level ≥ 100mg/dL or diagnosis of diabetes), dyslipidemia(take a medication).
